# Hearing Loss and Cognition: The Role of Hearing Aids, Social Isolation and Depression

**DOI:** 10.1371/journal.pone.0119616

**Published:** 2015-03-11

**Authors:** Piers Dawes, Richard Emsley, Karen J. Cruickshanks, David R. Moore, Heather Fortnum, Mark Edmondson-Jones, Abby McCormack, Kevin J. Munro

**Affiliations:** 1 School of Psychological Sciences, University of Manchester, Manchester, United Kingdom; 2 Centre for Biostatistics, Institute of Population Health, University of Manchester, Manchester, United Kingdom; 3 Population Health Sciences and Ophthalmology and Visual Sciences, School of Medicine and Public Health, University of Wisconsin, Madison, Wisconsin, United States of America; 4 Cincinnati Children’s Hospital Medical Center and Department of Otolaryngology, University of Cincinnati College of Medicine, Cincinnati, Ohio, United States of America; 5 NIHR Nottingham Hearing Biomedical Research Unit, Nottingham, United Kingdom; 6 MRC Institute of Hearing Research, Nottingham, United Kingdom; 7 Otology and Hearing Group, Division of Clinical Neuroscience, School of Medicine, University of Nottingham, Nottingham, United Kingdom; 8 Central Manchester Universities Hospitals NHS Foundation Trust, Manchester, United Kingdom; ARC Centre of Excellence in Cognition and its Disorders (CCD), AUSTRALIA

## Abstract

Hearing loss is associated with poor cognitive performance and incident dementia and may contribute to cognitive decline. Treating hearing loss with hearing aids may ameliorate cognitive decline. The purpose of this study was to test whether use of hearing aids was associated with better cognitive performance, and if this relationship was mediated via social isolation and/or depression. Structural equation modelling of associations between hearing loss, cognitive performance, social isolation, depression and hearing aid use was carried out with a subsample of the UK Biobank data set (n = 164,770) of UK adults aged 40 to 69 years who completed a hearing test. Age, sex, general health and socioeconomic status were controlled for as potential confounders. Hearing aid use was associated with better cognition, independently of social isolation and depression. This finding was consistent with the hypothesis that hearing aids may improve cognitive performance, although if hearing aids do have a positive effect on cognition it is not likely to be via reduction of the adverse effects of hearing loss on social isolation or depression. We suggest that any positive effects of hearing aid use on cognition may be via improvement in audibility or associated increases in self-efficacy. Alternatively, positive associations between hearing aid use and cognition may be accounted for by more cognitively able people seeking and using hearing aids. Further research is required to determine the direction of association, if there is any direct causal relationship between hearing aid use and better cognition, and whether hearing aid use results in reduction in rates of cognitive decline measured longitudinally.

## Introduction

The prevalence of dementia in those aged over 60 years is between 5–7%, with the numbers of those affected globally forecast to double every 20 years between 2010 and 2050 [1]. Cognitive decline and dementia have a profound impact on the individual, on caregivers and society, and the financial costs of cognitive decline and dementia are a major source of concern [2]. However, there is some cause for optimism in the form of potentially modifiable risk and protective factors, including cardiovascular health, psychological and emotional health, cognitive and physical activity, smoking and diet [3,4], and these may offer avenues for prevention. In this study we suggest that remediation and/or prevention of hearing loss may offer an additional avenue for prevention.

Hearing loss is common in older adults [5] and is associated with cognitive decline and incident dementia [6–11]. There are two main explanatory hypotheses for this association. The first is that the association between cognitive and hearing variables reflects a ‘common cause’, namely age-related changes in the nervous system. In this model, hearing loss and cognitive decline share common, age-related neurodegenerative mechanisms [8,11]. The second is the ‘cascade’ hypothesis, where long-term deprivation of auditory input may impact on cognition either directly, through impoverished input, or via effects of hearing loss on social isolation and depression [6,12,13]. Hearing loss is independently associated with social isolation and depression [14,15], and social isolation and depression are associated with cognitive decline [3,16,17]. One further possibility is that hearing impairment results in increased compensatory mental effort to perform cognitive tasks (such as remembering sequences of spoken digits [18]). This compensatory effort may use up limited cognitive resources resulting in an apparent decrement in cognition (the ‘cognitive load’ hypothesis [11]). However, this hypothesis seems unlikely to fully account for the association between hearing and cognitive performance given that the association between hearing and cognition remains similar whether cognition is tested with visual or auditory stimuli [10].

There is evidence that intervention in the form of hearing aids may improve quality of life and increase social engagement [19] and inconsistent evidence that hearing aid use may have a positive impact on performance of cognitive measures over a few weeks or months [20]. Some of the cognitive measures in these previous studies were auditory-based, so improvements may be due to improved audibility. In terms of longer-term outcomes of hearing aid use on cognition, Valentijn and colleagues [21] found no impact of sensory intervention (cataract surgery; n = 22 or hearing aids; n = 7) on cognitive measures 6 years after baseline. There is currently little evidence that hearing aids have a long term protective effect against cognitive decline.

The aim of this study was to model statistical associations between hearing impairment and cognitive performance in a large and inclusive data set. A positive association between hearing ability and cognitive performance could be consistent with both the cascade and common cause hypotheses. However, if auditory deprivation contributes to cognitive decline, as suggested by the cascade hypothesis, use of hearing aids should be associated with better cognitive performance. The mediating role of social isolation and/or depression was also investigated.

## Methods

### UK Biobank sample

UK Biobank was established for prospective investigations of the genetic, environment and lifestyle causes of diseases of middle and older age [22]. Ethical approval was obtained from the National Health Service North West Multi-centre Research Ethics Committee. More than 500,000 UK adults were tested between 2006–2010. Recruitment was via the UK National Health Service, and aimed to be as inclusive and representative as possible of the general population. Participants attended an assessment centre and gave informed consent. They completed a two hour test session that included a computerised assessment of lifestyle, environment and medical history, cognitive capacity and hearing. Information on the procedure and the additional data collected can be found elsewhere (http://www.ukbiobank.ac.uk/). All data were anonymized and de-identified prior to analysis. As UK Biobank data collection proceeded, additional measures were included for a subset of participants. Participants in the present study were a subset of 164,770 who were asked to complete a hearing test (the Digit Triplet Test).

### Demographic data

Sex, ethnicity data (based on 2001 UK Census categories) and Townsend deprivation score (based on the area of residence) were collected for each participant. Townsend deprivation scores are widely used in health studies as a proxy for socioeconomic status [23]. Lower Townsend scores represent areas associated with less deprived (i.e. more affluent) socioeconomic status. Participants were asked to rate their health with two self-report questions “In general how would you rate your overall health?” (excellent/good/fair/poor/do not know/prefer not to answer) and “Do you have any long-standing illness, disability or infirmity?” (yes/no/do not know/prefer not to answer).

### Digit Triplet Test

The Digit Triplet Test (DTT) is a speech-in-noise test originally developed in Dutch for reliable large scale hearing screening, and which correlates highly (*r* = 0.77) with audiometric thresholds [24,25]. The English version of the DTT used in the UK Biobank was developed at the University of Southampton [26] (for a demonstration, see http://www.actiononhearingloss.org.uk/your-hearing/look-after-your-hearing/check-your-hearing/take-the-check.aspx). The DTT procedure is described elsewhere (http://biobank.ctsu.ox.ac.uk/crystal/label.cgi?id=100049). Briefly, the signal to noise ratio (SNR), reported in decibels, for the 50% correct speech recognition threshold was estimated for each ear. The level of hearing loss was based on better ear performance. Hearing aid users performed the DTT without hearing aids.

### Cognitive tests

Cognitive tests were completed via a computerised touch screen interface. Further information is reported elsewhere (http://biobank.ctsu.ox.ac.uk/crystal/label.cgi?id=100026). Hearing loss would not be expected to contribute to performance on these visually presented tests. The background and rationale for the cognitive tests is reported by UK Biobank elsewhere (http://www.ukbiobank.ac.uk/wp-content/uploads/2011/11/UK-Biobank-Protocol.pdf?phpMyAdmin=trmKQlYdjjnQIgJ%2CfAzikMhEnx6).

#### Reaction time

This test was based on the card game ‘Snap’. Participants were shown two cards at a time, with 12 pairs of cards overall. If both cards display a matching symbol, participants pressed a response button with their dominant hand as quickly as possible. The outcome measure was the average time to correctly respond to a matching pair.

#### Pairs matching

Participants were asked to memorise the location of as many matching pairs of cards as possible. Cards were then turned face down, and the participant was asked to match as many pairs as possible with the fewest attempts. This test was presented in two rounds. The first round contained one set of cards in a 2x3 matrix with 3 matching pairs, the second round contained two sets of cards in a 3x4 matrix with 6 matching pairs. The outcome measure was the number of incorrect matches across all three sets.

#### Fluid intelligence

Fluid intelligence (the capacity for logical thought and problem solving, independent of acquired knowledge) was based on multiple choice responses to 13 questions such as “"Bud is to Flower as Child is to?" Participants had 2 minutes to complete as many questions as possible. Questions that were not completed within the time limit were scored as zero. The outcome measure was the sum of the number of correct answers.

### Hearing aid use, social isolation and depression

Hearing aid use was assessed via response to the question “Do you use a hearing aid most of the time?” Social isolation was assessed via response to the question “Do you often feel lonely?” Participants had the response options Yes/No/Do not know/Prefer not to answer. Depression was measured via response to the screening question; “Over the past two weeks, how often have you felt down, depressed or hopeless?” [27]. Participants had the response options Not at all/Several days/More than half the days/Nearly every day/Do not know/Prefer not to answer. Responses between ‘not at all’ and ‘nearly every day’ were scored from 1 to 4.

### Data analysis

Structural equation modelling [28] was used to test whether the association between hearing impairment and cognition may be mediated by hearing aid use, social isolation and/or depression in a sequence of four models, described in the Results. Structural equation modelling allows statistical evaluation of inter-relationships (pathways) between hearing impairment, cognition, hearing aid use, social isolation and depression while simultaneously controlling for the potential confounders of age, sex, general health and socioeconomic status. Structural equation modelling is a regression-based technique that requires data to be distributed along the range of variables (e.g. both hearing aid use and non-use, good to poor hearing). We considered mediation to be present when both the pathways constituting the indirect effect are statistically significant, and that this is partial mediation if the direct effect is also significant.

Cognition was measured by a standardised latent factor (mean 0, variance 1) in the structural equation model which was derived from a measurement model with observed indicators of the reaction time, pairs matching and fluid IQ tests. The covariates age, sex, general health (overall health rating and long-standing illness, disability or infirmity) and socioeconomic status (Townsend index) were included as predictors for each outcome variable in the overall structural equation model. Modelling was carried out using robust weighted least squares (WLSMV) in the Mplus program version 7.11 (*www.statmodel.com/*). Fit statistics and standardised coefficients were reported for each model. The Mplus estimates for paths from predictors to an observed categorical dependent variable (such as HA use and social isolation) are probit regression coefficients. A positive sign means that the probability of the categorical dependent variable (e.g. the category 1 for a 0/1 variable) is increased when the predictor value increases. A larger magnitude means that this probability is higher. For the standardised latent cognition variable a higher score implies worse cognition due to the direction of the factor loadings. The depression variable with four response levels was treated as a continuous variable. Estimates for paths from predictors to these dependent variables can be interpreted as in a standard linear regression.

## Results

Table 1 contains the sex, ethnicity and Townsend deprivation score for the subset included in the present study compared to the corresponding section of the UK population aged 40 to 69 years. The study sample contains a slightly higher proportion of females and people living in more affluent areas than in the general population. The proportion of White ethnicity is similar to that in the general population.

**Table 1:** Participants in the study sample versus 2001 UK Census data for sex, ethnicity and socio-economic status.

		UK Biobank	UK Census 2001
**Sex**	Male	45.5%	49.2%
**Ethnicity**	White	91.5%	91.3%
**Socioeconomic status**	Mean Townsend score* (SD)	-1.1 (2.9)	0.7 (4.2)

*Lower Townsend scores indicate less deprivation.

Sex and ethnicity are shown as percentages while socio-economic status is reported as average Townsend deprivation index score (with standard deviation).

**Fig 1:**
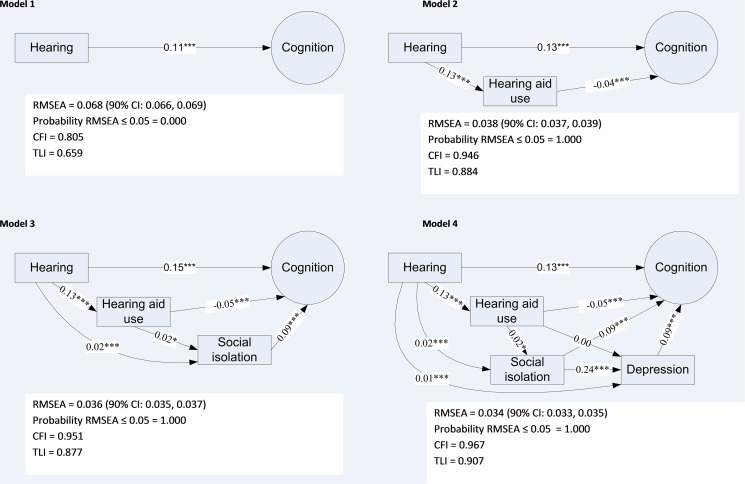
Structural equation models of standardised path coefficients between hearing, cognition, hearing aid use, social isolation and depression. Notes: ***p<0.001, *p<0.05. Root Mean Square Error of Approximation (RMSEA). A value less than 0.05 indicates good fit. Comparative Fit Index (CFI) and Tucker Lewis Index (TLI), with a number greater than 0.95 indicating good fit.

In Model 1, after controlling for age, sex, SES, and general health, poorer hearing remained significantly associated with poorer cognition (Fig. 1). However, despite each predictor being statistically significant, the model fit statistics indicated that the model was not satisfactory in explaining variation in cognition. In Model 2, for equivalent levels of hearing loss, hearing aid use was associated with better cognitive performance, supporting the cascade hypothesis. The effect of hearing loss on cognition remained significant, implying that the effect of hearing loss on cognition is only partly mediated through hearing aid use. Social isolation was associated with both poorer cognition and poorer hearing (Model 3), but hearing aid use was weakly associated with more social isolation. The effect of hearing aid use on cognition is partly mediated through social isolation, but there remains a significant direct effect. In Model 4, social isolation and poor hearing were significantly associated with higher frequency of depression. Frequency of depression and social isolation were associated with poorer cognition. Hearing aid use was not associated with depression, but was associated with greater social isolation and with better cognition. With the exception of the Tucker Lewis Index (TLI), fit statistics indicated that models 2–4 were a good fit with the data. As a sensitivity analysis and to provide a check of the robustness of the models, models 3 and 4 were re-run with alternative measures of depressive symptoms (frequency of unenthusiasm/disinterest) and social isolation (number of social/leisure activities). Use of alternative measures did not change the substantive results in either model (data not reported here).

## Discussion

In cross-sectional modelling in a large sample of UK adults, hearing aid use was associated with better cognition. This is consistent with the ‘cascade hypothesis’, where long-term auditory deprivation or degraded auditory input may result in increased cognitive decline [6,12,13]. The positive association of hearing aid use on cognition that was observed in the present study was independent of any positive association of hearing aid use on social isolation or depression. Therefore any effect of hearing aid use on cognition is unlikely to be via reduction of the adverse effects of hearing loss on social isolation or depression. Rather, these data suggest that the benefit may be directly through increased audibility of sounds in daily life. This pattern of association was observed within a large and inclusive sample of UK adults in the present study, and is likely to be generalisable to the UK population [22]. The cognitive tests were all visually presented, and so it is unlikely that hearing aids had a strong impact on performance on cognitive tests via improved audibility of test stimuli.

If hearing aids do have a positive impact on cognitive performance not due to a reduction in depression or social isolation, how might hearing aid use impact on cognition? According to the cascade hypothesis, untreated hearing loss may result in long-term auditory deprivation or degraded auditory input, resulting in increased cognitive decline. However, the mechanism for this is not known and requires elucidation [6,12,13]. One possibility is that hearing aids may boost self-efficacy, and increased self-efficacy positively impacts on performance on cognitive tests. Self-efficacy refers to the belief in one’s own ability to perform tasks and achieve goals. Hearing loss is associated with reduced self-efficacy [29]. Low self-efficacy is associated with poor performance on a variety of challenging tasks, perhaps via affective or motivational influences [30,31]. Unfortunately, no self-efficacy data were available in the present study and we were unable to examine this possibility.

One unexpected result was a lack of association between hearing aid use and depression, and increased social isolation associated with hearing aid use. It may be that hearing aids do discourage participation in social events by amplifying aversive background noise that is typical at social venues such as clubs, cafes and restaurants. However, hearing aids have been previously suggested not only to reduce hearing handicap, but to reduce concomitant social isolation and depression [32]. Evidence for this is limited however [33]. One randomised controlled study reported an improvement in social engagement and a small reduction in symptoms of depression in a select group of new hearing aid users (elderly white male US veterans with moderate to severe hearing loss) [19]. An explanation for the lack of positive association between hearing aid use and social isolation in the present study might be that the measure of social isolation based on a single Yes/No question lacked sensitivity. Note however, that associations remained unchanged when substituting an alternative measure of social engagement. Similarly with depression, associations were similar for an alternative measure of depressive symptoms. Information about hearing aid use was limited to whether participants reported that they use a hearing aid ‘most of the time’. The amount of hearing aid use, how well the hearing aid was fitted to compensate for hearing loss, the duration of hearing aid use and whether participants began using hearing aids soon after the onset of hearing loss may also impact the effectiveness of hearing aids in improving outcomes including social engagement, depression and cognition [34]. However, one would expect that in a sample of the size utilized in the present study, the net effect of hearing aid use on social engagement, depression and cognition would be apparent.

The assumption in the present study was that better cognition in hearing aid users observed in cross-sectional analysis may reflect the long-term impact of hearing aid use in reducing cognitive decline. However, longitudinal data are required to confirm whether hearing aid use is associated with any alteration in the rate of cognitive decline over time. The data in the present study are correlational, and no strong conclusions about causality are possible. Alternative interpretations of the patterns of association reported in the present study are possible. For example, rather than hearing aids ‘causing’ better cognition, cognitively more able people might tend to obtain and use hearing aids. Cognitively more able people may be more likely to access specialist health services, including audiology, or may more likely recognise hearing disability and seek treatment. Establishing a causal association between hearing aid use and cognitive performance requires controlled studies with cognitive outcomes measured in the short term as well as after several years hearing aid use. The study was restricted to adults aged 40 to 69 years, so it is uncertain whether the associations identified in the present study are generalizable to older adults, in whom sensory impairment, hearing aid use and cognitive impairments are more common.

## Conclusion

Hearing aid use was associated with better cognition in a large cross-sectional study of UK adults. The association was independent of social isolation and depression. Further research is required to determine the direction of association, if there is any direct causal relationship between hearing aid use and better cognition, and whether hearing aid use results in reduction in rates of cognitive decline measured longitudinally. Treating hearing loss may make a significant contribution to reducing the burden associated with cognitive decline and reduced quality of life.
